# Vaccine Hesitancy in College Students

**DOI:** 10.3390/vaccines11071243

**Published:** 2023-07-15

**Authors:** Emily Gilbert-Esparza, Amelia Brady, Sierrah Haas, Heather Wittstruck, Jennifer Miller, Qing Kang, Ellyn R. Mulcahy

**Affiliations:** 1 Kansas Department of Health and Environment, Topeka, KS 66612, USA; 2 Master of Public Health Program, College of Veterinary Medicine, Kansas State University, Manhattan, KS 66506, USA; 3 Department of Statistics, Kansas State University, Manhattan, KS 66506, USA

**Keywords:** vaccine hesitancy, COVID-19, college students, public health, vaccines

## Abstract

(1) Background: Following the COVID-19 pandemic, vaccine hesitancy has become an increasingly important topic and has created significant concerns in public health. It is important to explore vaccine hesitancy among college students as they have been identified to be a high-risk group for COVID-19 transmission. This study aims to investigate COVID-19 vaccine hesitancy in college students on a midsized midwestern university campus. (2) Methods: Data were collected from 311 undergraduate and graduate college students during June and July 2021. Participants completed a survey on COVID-19 vaccine behaviors, perceptions, and opinions. Quantitative and qualitative analysis was performed to identify vaccine hesitancy and influencing factors in the student population. (3) Results: The results of this study demonstrated significant relationships between older and younger undergraduate students (OR > 1, *p* < 0.05), students who received a yearly influenza vaccine and those that did not (*p* < 0.05), and students who had a previous COVID-19 infection and those that did not (OR > 1, *p* < 0.05). We also determined a significant difference between some racial/ethnic groups and vaccine hesitancy status. (4) Conclusions: COVID-19 vaccine hesitancy exists on college campuses, and is influenced by age and student status, influenza vaccination status, previous COVID-19 infection, and race/ethnicity.

## 1. Introduction

### 1.1. Vaccine Hesitancy

Vaccine hesitancy is a growing public health concern in the United States and worldwide [1]. The World Health Organization (WHO) lists vaccine hesitancy as one of the top ten threats to global health and defines it as a delay in vaccination or a lack of acceptance of vaccines [2,3]. In a 2018 survey performed by the WHO and United Nations Children’s Fund Joint Reporting Form, 74% out of the 194 countries surveyed listed vaccine hesitancy as a public health concern in their country [4]. Vaccine hesitancy is not limited to developing countries; countries with a higher gross domestic product (GDP) have been found to have the lowest confidence levels in vaccines [5]. Vaccine hesitancy not only has public health consequences but economic consequences as well. For example, misinformation and disinformation about COVID-19 vaccines caused an estimated USD 50 to 300 million worth of harm every day in 2021 [5].

### 1.2. Impact of COVID-19

In the wake of the COVID-19 pandemic, vaccine hesitancy has become an important topic and has created significant concerns in public health. As of 1 February^,^ 2023, the United States has had over 1 million deaths from COVID-19, with weekly new deaths averaging around 4000 individuals each week [6]. If these trends continue, COVID-19 will remain the leading cause of death in the United States [7]. An important population to consider for COVID-19 impact is college students. Vaccine hesitancy among college students has consequences at both individual and collective levels. Individually, it puts students at risk of contracting and spreading vaccine-preventable diseases, leading to personal health complications and academic disruptions. Collectively, it increases the likelihood of outbreaks on campus, straining healthcare resources and impacting the broader campus community. Additionally, collective vaccine hesitancy weakens herd immunity and compromises the protection of vulnerable populations. Approximately 40% of 18- to 24-year-olds enroll for college with 43% of high school completers enrolling immediately after graduating [8]. It is estimated that 19.4 million United States adults are enrolled in postsecondary education [9].

### 1.3. COVID-19 Impact on College Students

Of COVID-19 cases in the United States, individuals on college campuses account for more than 397,000 cases, making college students an important part of the disease transmission network [7]. Young adults are less likely to get vaccinated, with 48% of college students surveyed reporting COVID-19 vaccine hesitancy [10]. In November 2021, a study conducted at a midwestern university confirmed that college students may be more hesitant to receive COVID-19 vaccinations with 50% of the survey population not vaccinated. Results showed that out of the unvaccinated population, 49% had no intention to be vaccinated [11].

College students are important to consider when it comes to disease transmission for several reasons. Due to the nature of their activities, college students can have the ability to become “super spreaders,” where they pass on the virus to an unusually or unexpectedly large number of individuals [12]. Many college students have close or crowded living arrangements. Furthermore, they engage in social activities on and off campus where many people are present, leading to an increased risk of infecting others [12]. College students are also often employed in locations where they may come into contact with the general public, creating a potential transmission link between students and the rest of the community [12]. For many college students, university breaks are a time to travel home to visit family and friends, whether that is local or international. Travel heightens concerns regarding pathogen transmission as it brings together individuals with varying vaccination statuses and increases the likelihood of encountering infectious diseases [10].

### 1.4. Vaccine Hesitancy in College Students

To combat vaccine hesitancy, we must discover how and why it occurs. It is a common belief that vaccine-hesitant individuals are against science or do not understand it; however, that is not necessarily true. A study to determine why college students refuse vaccines showed that participants who did not intend to receive the COVID-19 vaccine were more fearful of the potential side-effects, the US government, the safety of the COVID-19 vaccine, and the efficacy of the COVID-19 vaccine [12]. This study also found that college students commonly cited hearing or reading negative media about the COVID-19 vaccine as a reason for hesitancy [12]. An interesting aspect of vaccine hesitancy is the concept that personal experience outweighs other evidence. Adverse events can impact an individual’s vaccine experience or beliefs. Although most adverse events are coincidental, people believe they are related since they happen so close to receiving vaccinations [1]. Lacking trust in the physician can occur for various reasons and can add to vaccine hesitancy. It has been shown that mistrust in conventional medicine is a strong indicator of vaccine hesitancy [2].

During the COVID-19 pandemic, many different reasons for vaccine hesitancy have emerged. For college students, making vaccination decisions may be the first independent medical decision they have ever made. In a study performed on a college campus regarding the influenza vaccine, 55% of undergraduates were not vaccinated and 56% said their parents usually made their medical decisions [12]. Graduate students had a higher percentage of vaccinations (72%), and only 23% relied on their parents for medical decisions [12]. Some groups that have the highest percentages of vaccine hesitancy are multiracial students and students who are not first-generation college students [12]. Other reasons for which the students stated that they did not get vaccinated were low accessibility to the vaccine and a lower perceived risk of contracting influenza [13]. Other general factors contributing to this hesitancy vary widely and include concerns about vaccine safety, efficacy, side-effects, or a general lack of trust in the healthcare system. Misinformation and conspiracy theories circulating on social media platforms have also played a role in shaping students’ perceptions [14].

Studies have discovered that socioeconomic factors also play a significant role in vaccine hesitancy. Socioeconomic factors such as limited access, affordability, health disparities, educational background, peer influence, political beliefs, and lack of trust in institutions contribute to vaccine hesitancy among college students. These factors can shape students’ perceptions, beliefs, and decisions regarding vaccination. [15,16]

### 1.5. Strategies for Effective Communication on Vaccination

College students routinely use online platforms and information technology to make decisions, especially during the COVID-19 pandemic [17]. One study found that 60% of college students receive their health information from social media [7]. Another study found that college students looking for health information on social media are influenced by several aspects including the usefulness of information and people and subjective norms [18]. College students are found to use social media as their preferred source of health information [18]. It has also been shown that low health literacy is correlated with confusion about the information found on the internet, whereas high health literacy correlates with the use of more trustworthy, reputable web-based information and less fear of COVID-19 [17].

The WHO lists six determinants of trust that can be used to help combat this problem: competence, objectivity, fairness, consistency, sincerity, and faith [19]. When these six determinants of trust are incorporated into relevant and specific educational materials, tailored to specific audiences, and include information about the benefits and risks of vaccination, vaccine trust can be increased [19]. For instance, on college campuses, university administrators, campus housing leaders, athletic directors, and student organization leaders can be used to positively influence vaccine acceptance [19]. Student-led groups on college campuses can effectively influence vaccine acceptance in a student body by providing clear and concise positive messaging as well as utilizing university-sponsored social media outlets [10]. Reinforcing the message of community protection through these leaders has had a positive impact [4]. Vaccine information (or health information in general) should be clear and to the point, easy to access, user-friendly, relevant, easy to understand, and culturally appropriate to be most effective [6,20].

Effective strategies in addressing vaccine hesitancy include but are not limited to providing accurate information through educational campaigns, leveraging peer influence and role models, ensuring the accessibility and convenience of vaccines, personalizing communication, engaging student organizations, fostering dialogue to address concerns, promoting cultural competence and inclusion, collaborating with faculty and staff, and monitoring and countering misinformation. These strategies aim to educate, engage, and empower students to make informed decisions about vaccination and contribute to a healthier campus community [21].

Overall, vaccine hesitancy is a complex, global issue. College students report many reasons for vaccine hesitancy, from personal experience to mistrust of the government. Therefore, a single, one-size-fits-all solution to the growing problem does not exist. Instead, public health leaders must consider tailoring a strategy specifically for college students to address vaccine hesitancy for their population. Increasing knowledge and health literacy, clear communication that includes listening to the concerns of students, and using vaccine advocates that the population will trust are crucial components to addressing vaccine hesitancy.

## 2. Materials and Methods

Prospective participants were recruited to voluntarily participate in the study via email distributions and via a daily campus newsletter sent to all students on a midsized midwestern university campus from 9 June to 13 July 2021. A link to the informed consent and the study survey was placed directly in the newsletter announcement and the distributed e-mail message. Currently enrolled undergraduate and graduate students on the university campus who were interested in participating accessed the study via the link to an online survey developed in Qualtrics, which was accessible on any internet-enabled device. The survey was issued via Qualtrics, with the ExpertReview response quality feature utilized for quality checks, including for prevention of duplicates, identification of potential speeders (respondents who complete the survey too quickly), identification of incomplete responses, and identification of responses in progress. Two reminders were sent to facilitate the completion of responses in progress.

Prior to the collection, participants provided informed consent and were asked to provide demographic information, including student status as undergraduate or graduate status, race/ethnicity, age, and family income, in addition to answering 14 questions related to vaccine hesitancy, with both quantitative and qualitative answers. Three questions had two possible answer choices, three questions had three possible answer choices, seven questions had four possible answer choices, two questions had six possible answer choices, and one question had seven possible answer choices. Participants were able to choose more than one answer for five questions. Four questions had the option of text entry.

The study protocol was approved by the institutional review board (IRB #10719) of the university and informed consent was obtained for all participants. This study draws from data collected as part of a master’s degree report and extends the analysis to identify recurring patterns and emerging themes that are relevant to vaccine hesitancy in the context of public health. The survey questionnaire and sampling methodology described in this study were developed specifically for the master’s report [22].

### 2.1. Data Analysis

#### 2.1.1. Quantitative Analysis

Vaccine hesitancy status was determined using the question “Will you receive a COVID-19 vaccine?” with four response levels: 1 (“No”), 2 (“Will wait”), 3 (“Will receive”), 4 (“Have received”). This ordinal response was analyzed using cumulative logistic regression models. The explanatory variable was assumed to have a constant multiplicative effect on the odds of cumulative vaccine hesitancy, including level£1, level£2, and level£3. The model goodness of fit was verified by assuring the ratio of deviance to degrees of freedom was no greater than two.

Explanatory variables age and student status were associated (Pearson’s chi-square *p*-value < 0.001; Phi coefficient = 0.51). To avoid Simpson’s paradox, these two variables and their interaction were modeled together. There were no undergraduate students in the 27 and older group who selected 4 (“Have received”). The simple regression model with the annual flu-shot vaccination had a poor fit (deviance-over-degree-of-freedom ratio was 6.45). In the meantime, the association of the annual flu-shot vaccination was noticed with age (Pearson’s chi-square *p*-value = 0.020; Phi coefficient = 0.13) and student status (Pearson’s chi-square *p*-value ≤ 0.001; Phi coefficient = 0.21). There were no undergraduate students in the 27 and older group who responded “yes” to getting an influenza vaccine every year. To overcome overdispersion, the present work performed the multiple regression analysis where the three variables and all their two-way interactions were in the model. The three-way interaction was not estimable, because of a missing combination. The effect of interactions was evaluated via the type 3 likelihood-ratio (LR) chi-square test.

For the rest of the demographic characteristics, their association with COVID-19 vaccine hesitancy was examined separately. Family income and history of COVID-19 tests were collected via single-choice questions and were analyzed using the simple regression model. Race/ethnicity, source of information for COVID-19 vaccine, and experience of negative health effects due to COVID-19 were multiple-choice questions. Each of the choices corresponded to a binary explanatory variable. They were analyzed using the multiple logistic regression model. Their overall effect was evaluated via the global test using the LR chi-square statistics. There were no students who selected Native Hawaiian or Other Pacific Islander.

The estimated cumulative probabilities of COVID-19 vaccine hesitancy and their standard errors (SEs) were reported for every level of demographic characteristics. Pairwise comparisons among levels of demographic characteristics were performed based on the two-sided LR chi-square test for non-zero differences in log-cumulative odds. Statistical tests were performed at the 0.05 level. No multiplicity adjustment was applied. Appendix A provides a full report of all statistical procedures performed.

Attitude toward vaccine safety and efficacy were collected using the questions: “How confident are you that the COVID-19 vaccine is safe?” and “How confident are you that the COVID-19 vaccine is effective?” with three response levels: 1 (“Not confident”), 2 (“Undecided”), 3 (Confident”). Attitude toward vaccine mandate was collected using the question “Should universities require the COVID-19 vaccine in order to attend in future semesters?” with three response levels: 1 (“No”), 2 (“Undecided”), 3 (“Yes”). There were no students in the not-vaccinated group who selected 2 (“Undecided”) or 3 (“Yes”). The association of these ordinal variables with vaccine hesitancy was measured using Kendall’s Tau-b and Stuart’s Tau-c. Both measurements are on the −1 to 1 scale with values close to 1 being highly concordant (strongly positively associated), and values close to −1 being highly discordant (strongly negatively associated). SAS statistical analysis was executed via Statistical Analysis Software (SAS version 9.4; Cary, NC) LOGISTIC and GENMOD procedures.

#### 2.1.2. Qualitative Analysis

The written qualitative answers were reviewed immediately after the survey closed and were examined for themes of vaccine hesitancy and non-hesitancy for both COVID-19 and influenza. In total, 47 separate written answers were evaluated and two were removed due to ambiguous text or phrasing, leaving a total of 45 to be examined. The answers were read again independently, and codes were assigned for qualitative analysis of thematic content, with themes related to vaccine hesitancy and non-hesitancy. To assure accurate coding of the data, the authors discussed and confirmed agreement for the identified recurring patterns and emerging themes. The corrected, typed transcripts and notes were entered into NVivo12 Plus (Version 1.5.1; QSR International LTD, 2018) to classify, sort, and analyze the data. Five major hesitancy themes were developed: concern of health risks, perception of not being at risk, mistrust of the vaccine, prior infection, and concern of vaccine long-term effects. The major non-hesitancy themes developed were work- or school-related, a wish to return to normal, moral obligation, protecting myself and others, trust in the vaccine, and trust in the research process.

## 3. Results

In the spring 2021 semester, a survey was conducted with students at a midwestern university. A total of 345 responses were received for the survey. Of those, seven did not complete the survey, one had unknown student status, and twenty-six were not students. This left a remainder of 311 completed responses, including 132 undergraduate students and 179 graduate students. Table 1 shows the student status and race/ethnicity of the population of this study.

Table 2 presents the distribution of COVID-19 vaccination status by age and student status. Notably, undergraduates in the 27 and older age group exhibited higher vaccine hesitancy compared to undergraduates in the 18–26 age group (OR > 1, *p* = 0.002). No student in this group selected “have received.” Graduate students in the 18–26 age group showed a tendency toward higher vaccine hesitancy than those in the 27 and older age group, but the difference was not significant (*p* > 0.05).

Table 2 also presents the results of COVID-19 vaccination status stratified by race and ethnicity. American Indian or Alaska Native students were more likely to be hesitant than Asian students and Black or African American students (OR > 1, *p* = 0.016). There was a difference between American Indian or Alaska Native students compared to Hispanic or Latino students and White students, but it was not statistically significant (OR > 1, *p* > 0.05). Asian students were less likely to be hesitant than White students (OR < 1, *p* = 0.003), and Hispanic or Latino students, but this was not statistically significant (OR < 1, *p* > 0.05). Asian students were more likely to be hesitant than Black or African American students, but it was not statistically different (OR > 1, *p* > 0.05). Black or African American students were less likely to be hesitant than Hispanic or Latino students (OR < 1, *p* = 0.023) and White students (OR < 1, *p* = 0.007). No difference was seen in hesitancy between Hispanic or Latino students and White students. No students self-identified as Native Hawaiian or Other Pacific Islanders.

The impact of influenza vaccination on COVID-19 vaccine hesitancy was also examined. Students who received an annual influenza vaccine were less likely to be hesitant toward the COVID-19 vaccine (*p* = 0.0119).

Additionally, a higher likelihood of vaccine hesitancy was observed among students with a previous COVID-19 infection compared to those without a previous infection (OR > 1, *p* = 0.038).

Sources of information used by students on COVID-19 vaccine hesitancy did not have a significant effect on vaccine hesitancy (OR = 1, *p* > 0.05).

Finally, an evaluation was conducted to assess the factors contributing to vaccine hesitancy among students. Table 3 shows vaccine hesitancy themes that were identified by qualitative analysis of three survey questions. The most frequently recurring themes in these answers were concern about health risks, perception of not being at risk, mistrust of the vaccine, prior COVID-19 infection, and concerns about the vaccine’s long-term effects. The remaining themes of no time availability (17), a non-effective vaccine (12), and considered themselves healthy (12) were each identified less than three times.

Table 3 also shows the COVID-19 vaccine non-hesitancy themes that were identified by qualitative analysis of one question from the survey. The most frequently recurring themes were work- or school-related, a wish to return to normal, moral obligation, and protecting myself and others. The remaining themes of disease prevention (17) and to avoid quarantine (12) were each identified less than three times.

## 4. Discussion

This study was conducted with the objective of examining the presence of COVID-19 vaccine hesitancy among college campuses and, if identified, investigating the underlying reasons for student hesitancy. The data presented indicate that undergraduates in the 27 and older age group were more likely to be vaccine-hesitant than undergraduates in the 18–26 age group. No undergraduates in the 27 and older age group selected “have received” for COVID-19 vaccination status. Examining the potential characteristics and demographics of undergraduates in the 27 and older age group population provides valuable insight into their hesitancy reasoning. Older undergraduates are non-traditional students who may have families of their own and are most likely not under the medical direction of their parents [20,23]. With families of their own, students may find it difficult to find time in their schedule to get vaccinated [24]. These students may have personal experiences with vaccines, physicians, or any part of the healthcare system that may affect their vaccination decisions [25]. These students are likely transitioning from the workforce back to school, so they may not have health coverage or the means to pay for out-of-pocket vaccinations [26]. We also saw that graduate students in the 18–26 age group were more likely to be hesitant than graduate students in the 27 and older age group but it was not statistically significant. Overall, the graduate students were less likely to be vaccine-hesitant than the undergraduate students. This is supported by previous studies that have shown that graduate students reported a higher percentage of vaccinations [11]. Reasons for this could include educational level, making independent medical decisions, and even holding positions where vaccinations are required [27].

This study revealed differences in COVID-19 vaccination status across various racial and ethnic groups. American Indian or Alaska Native students are more likely to be hesitant than Asian and Black or African American students, but not more likely to be hesitant than Hispanic, Latino, or White students. In prior studies, American Indian or Alaska Native college students were found to be twice as hesitant as White students regarding receiving the COVID-19 vaccine [27]. This is partially supported by the data presented since no statistically significant difference was seen between this group and Hispanic, Latino, or White students.

The study findings demonstrate a lower likelihood of vaccine hesitancy among Asian students compared to White students. Asian students are also less likely to be hesitant compared to Hispanic or Latino students, although it is not statistically different. Our data show that Asian students are more likely to be hesitant than Black or African American students but there is no statistical difference. In previous studies, Asian students were found to be half as hesitant than White students and less likely to be hesitant when compared with Black students, which contradicts our data [27].

Findings indicate a lower likelihood of vaccine hesitancy among Black or African American students compared to Hispanic, Latino, or White students. No difference was seen in hesitancy between Hispanic or Latino and White students, which contradicts previous studies. Previous data show Hispanic or Latino individuals as more hesitant (63.5%) than White individuals (46.2%). No students identified as Native Hawaiian or Other Pacific Islander, so no comparisons were made with that group.

When COVID-19 vaccination was examined by yearly influenza vaccination status, our data showed that students who receive an annual influenza vaccine are less likely to be hesitant to receive the COVID-19 vaccine. These data draw on a conclusion that if students are not hesitant toward the influenza vaccine, they would simultaneously not be hesitant toward the COVID-19 vaccination.

The investigation also aimed to understand the reasons behind students consistently receiving their influenza vaccine annually. Similar to the non-hesitancy observed for COVID-19 vaccination, students expressed non-hesitancy toward influenza vaccination due to factors such as the intention to protect oneself and others, trust in the vaccine’s effectiveness, confidence in the research process, and considerations related to work or school. Influenza vaccination status was shown to be associated with student status and age. We examined undergraduate and graduate students in the 18–26 age group and the 27 and older age group and whether they receive a yearly influenza vaccine in terms of COVID-19 vaccine hesitancy status. For undergraduate students, those in the 18–26 age group who receive an influenza vaccine every year are less likely to be hesitant to the COVID-19 vaccine than those who do not receive an influenza vaccine every year. In the undergraduates in the 27 and older age group, students who did not receive an influenza vaccine every year were more likely to be hesitant to the COVID-19 vaccine. We need to consider that this group who answered “no” only comprised eight participants. While this is not a large sample number for our study, if we used this model in a larger population size, we would expect to see these results. In this same group, no students responded “yes” to receiving the influenza vaccine every year, so we are not able to compare those responses. We did not find a difference in vaccine hesitancy in the graduate students in either age group whether they get an influenza vaccine every year or not.

The data reveals that students who have experienced a prior COVID-19 infection exhibit a higher likelihood of vaccine hesitancy compared to those without a previous COVID-19 infection. One reason for this may be because they have been infected with COVID-19, so they perceived that they do not need the vaccine. Prior COVID-19 infection may contribute to this altruistic thought that others need the vaccine more than them since they already have some natural protection. A study on influenza showed that many college students decline the influenza vaccine due to a low perceived risk of contracting the disease [13]. This could be of importance in terms of COVID-19 as well. Those individuals who have had a COVID-19 infection may think they are less likely to contract the virus again or that their natural immunity from the prior infection will protect them. This results in individuals not seeing value in receiving the vaccine.

The data indicate that while students are less likely to be hesitant to the COVID-19 vaccine when someone close to them has died from COVID-19 as compared to those having no negative health effects, the difference is not statistically different. No difference was seen in the other negative health effect groups. Participants had the following answer choices: they had negative health effects themselves, someone close to them had negative health effects, someone close to them died from COVID-19, or no negative health effects to themselves or someone close to them. With a total response number of 333, it is evident that some participants chose more than one answer. Even with more than one negative health effect, vaccine hesitancy was not affected. From the literature, we have seen that personal experience may outweigh other evidence and that intuition or experience is a powerful decision-making tool for college students [25]. So, we expected to see less hesitance in groups with negative health effects or death since the negative experience(s) with COVID-19 might have shaped their decision to get vaccinated.

When examining COVID-19 vaccination status by sources of information, our data show that sources of information did not affect COVID-19 vaccine hesitancy. Participants were able to choose all choices that applied, with a total of 647 responses to this question. This is important because it shows that students used a variety of sources and got their vaccine decision information from more than one source. Our data contradicts previously reported data that social media jeopardizes public health strategies and information [14]. Our study shows that information gathered online was no different than information obtained from a healthcare provider or peer-reviewed articles when it comes to vaccine hesitancy. While one study found that 60% of college students receive their health information from social media, our study shows that other sources of information were also researched and considered when making vaccine decisions [7].

Qualitative analysis allowed us to view vaccine hesitancy and non-hesitancy themes that were in the open-ended questions of our survey. The most frequent COVID-19 vaccine hesitancy themes identified were the perception of not being at risk (***n*** = 8), mistrust in the vaccine (***n*** = 7), and vaccine long-term effects (***n*** = 7) followed by the concern of health risks (***n*** = 5) and prior COVID-19 infection (***n*** = 5). Overall, these themes match those identified in other studies of overarching mistrust in the vaccine and government (2,10, 12, 15). The most frequent COVID-19 vaccine and influenza non-hesitancy themes identified were work- or school-related (COVID-19 ***n*** = 5, influenza ***n*** = 3), a wish to return to normal (COVID-19 ***n*** = 5), moral obligation (COVID-19 ***n*** = 6), to protect myself and others (COVID-19 ***n*** = 7, influenza ***n*** = 2), trust in the vaccine (influenza ***n*** = 2), and trust in the research process (influenza ***n*** = 2).

Our results highlight variations in vaccine hesitancy across different student groups, emphasizing the need for targeted interventions tailored to address the specific concerns and barriers faced by each group. For instance, age was found to be a significant factor, with higher hesitancy observed among certain age cohorts. To effectively address this, strategies should be developed to provide accurate information and tackle misconceptions specific to each age group. Furthermore, our study reveals racial and ethnic disparities in vaccine hesitancy, underscoring the importance of implementing culturally sensitive communication strategies and engaging trusted community leaders. By incorporating these recommendations into public health interventions, educational institutions and policymakers can contribute to reducing vaccine hesitancy and promoting higher vaccine uptake among college students. A continual monitoring and evaluation of vaccine attitudes and targeted interventions will be crucial for effectively addressing emerging concerns and promoting vaccine confidence within this population.

There were limitations in our study. Our data may contradict previous studies due to several factors. Our survey was given to students at a midsized midwestern college in a partially rural county. Our survey had 311 respondents, which is a smaller sample size compared to previous studies. Our sample was less racially and ethnically diverse with many of our respondents identifying as White (***n*** = 210). The other racial and ethnic groups had relatively low sample sizes compared to the White group, including American Indian or Alaska Native having only five responses and no responses recorded for Native Hawaiian or Other Pacific Islander. Also, some students identified as more than one race and ethnicity so they may be counted more than once.

Furthermore, the use of convenience sampling in this study may limit the generalizability of findings. Participation was based on accessibility and willingness, meaning that the target population may not be accurately represented. Future studies should consider more rigorous sampling methods.

Additionally, it is important to acknowledge that the study may have further limitations regarding the impact of pre-existing medical knowledge on vaccine hesitancy. Participants’ existing knowledge, or lack thereof, has the potential to influence their interpretation of information obtained from different sources. To gain a more comprehensive understanding, future surveys should consider differentiating between medical students and non-medical students, as well as students of different academic disciplines, as their baseline knowledge, and potential biases may vary.

Also notably important, in this study, was that the research was conducted during the spring 2021 semester. At the time of this research, all phase-two trial data on the available COVID-19 vaccines had not yet been released. This is a significant point to consider when interpreting the study’s results as a lack of trial data may have played a part in increased vaccine hesitancy [28,29,30]. The absence of phase-two trial data during the time of the study could have influenced the participants’ perceptions and attitudes toward the COVID-19 vaccine. Without the availability of efficacy data, individuals may have had less information about the vaccines’ effectiveness and safety profiles. This lack of data could have contributed to increased vaccine hesitancy or uncertainty among the participants.

Therefore, it is important to acknowledge that the findings of this study regarding vaccine hesitancy among college students may not fully capture the subsequent changes in attitudes and perceptions that may have occurred following the release of phase-two trial data. The evolving landscape of COVID-19 vaccine research and public discourse should be considered when interpreting the results and applying them to current circumstances.

Vaccine hesitancy is an important topic, especially as the COVID-19 pandemic continues. Many factors play into vaccine hesitancy and individual vaccine decision-making processes, and our survey sought to address some of these factors. We saw that age and student status, influenza vaccination status, previous COVID-19 infection, and race/ethnicity may affect vaccine hesitancy, while sources of vaccine information and whether the student or someone close to them had negative health effects or died from COVID-19 did not affect vaccine hesitancy.

## 5. Conclusions

Despite the existence of numerous publications addressing the issue of vaccine hesitancy among college students, this study offers a unique contribution by examining the topic from a fresh perspective. While previous studies may have explored similar themes, the novelty of this research lies in its focus on a distinct population. By conducting a survey specifically targeting college students, this study provides valuable insights into the factors influencing vaccine hesitancy within this specific demographic.

Our findings contribute to a better understanding of vaccine hesitancy among college students and provide insights that can inform public health interventions and strategies aimed at addressing vaccine hesitancy on university campuses. By identifying the specific factors influencing hesitancy and non-hesitancy, targeted interventions can be designed to increase vaccine acceptance and uptake among college students.

Overall, this study emphasizes the need for tailored approaches to address vaccine hesitancy among college students, taking into account age, student status, race/ethnicity, previous infection, and influenza vaccine acceptance. By addressing the concerns and motivations specific to this population, we can continue to enhance vaccine education, access, and uptake among college students, contributing to the collective effort to combat infectious diseases and promote public health.

Further research on vaccine hesitancy among college students is necessary to expand our understanding in this area. Longitudinal studies can provide insights into the changes in vaccine attitudes over time and evaluate the effectiveness of interventions aimed at reducing hesitancy. Investigating the role of social networks, peer influence, and the impact of tailored interventions within different student populations is an important area for future exploration. Additionally, examining the effectiveness of alternative communication strategies, such as social media campaigns or peer-led interventions, can contribute to promoting vaccine confidence and countering misinformation. Future research should also explore the long-term effects of interventions and evaluate their sustained impact on vaccine acceptance among college students.

## Figures and Tables

**Table 1:** Study population demographics.

Student Status	*n*	%
Undergraduate	132	42.4
Graduate	179	57.6
Total	311	100
Race/Ethnicity	***n***	**%**
White	210	66.5
Hispanic or Latino	29	9.2
Black or African American	15	4.7
Asian	57	18
American Indian or Alaska Native	5	1.6
Hawaiian or Other Pacific Islander	0	0

**Table 2:** COVID-19 vaccination hesitancy.

		COVID Vaccination Status, *n* (%)			
		No	Will Wait	Will Receive	Received
Student Status and Age	Undergraduate 18–26	36 (29%)	16 (13%)	9 (7%)	63 (51%)
	Undergraduate 27+	6 (75%)	1 (13%)	1 (13%)	0 (0%)
	Graduate 18–26	8 (10%)	2 (3%)	9 (11%)	61 (76%)
	Graduate 27+	4 (4%)	2 (2%)	7 (7%)	86 (87%)
	No	47 (26%)	16 (9%)	10 (6%)	105 (59%)
	Yes	7 (6%)	5 (4%)	15 (12%)	99 (79%)
Race and Ethnicity	American Indian or Alaska Native	2 (40%)	0 (0%)	0 (0%)	3 (60%)
	Asian	0 (0%)	3 (5%)	9 (16%)	45 (79%)
	Black or African American	1 (7%)	0 (0%)	0 (0%)	14 (93%)
	Hispanic or Latino	1 (3%)	4 (14%)	5 (17%)	19 (66%)
	Native Hawaiian or Other Pacific Islander	0 (0%)	0 (0%)	0 (0%)	0 (0%)
	White	48 (23%)	14 (7%)	13 (6%)	135 (64%)
Influenza Vaccine Status	No	47 (26%)	16 (9%)	10 (6%)	105 (59%)
	Yes	7 (6%)	5 (4%)	15 (12%)	99 (79%)
COVID-19 Infection	No	41 (16%)	18 (7%)	21 (8%)	180 (69%)
	Yes	13 (30%)	3 (7%)	4 (9%)	24 (55%)
Negative Health Effects	Self	4 (15%)	2 (8%)	3 (12%)	17 (65%)
	Someone close	15 (14%)	7 (7%)	8 (8%)	74 (71%)
	Someone close died	2 (7%)	2 (7%)	1 (4%)	23 (82%)
	No negative health effect	33 (19%)	13 (7%)	15 (9%)	114 (65%)
Vaccine Requirements	No	54 (43%)	16 (13%)	4 (3%)	53 (42%)
	Undecided	0 (0%)	3 (6%)	5 (10%)	40 (83%)
	Yes	0 (0%)	2 (2%)	16 (12%)	111 (86%)
Vaccine Efficacy	Not Confident	33 (69%)	5 (10%)	1 (2%)	9 (19%)
	Undecided	16 (30%)	7 (13%)	2 (4%)	28 (53%)
	Confident	5 (2%)	9 (4%)	22 (11%)	167 (82%)
Vaccine Safety	Not Confident	41 (73%)	5 (9%)	1 (2%)	9 (16%)
	Undecided	9 (17%)	8 (15%)	2 (4%)	35 (65%)
	Confident	4 (2%)	8 (4%)	22 (11%)	160 (82%)
Vaccine Requirements	No	54 (43%)	16 (13%)	4 (3%)	53 (42%)
	Undecided	0 (0%)	3 (6%)	5 (10%)	40 (83%)
	Yes	0 (0%)	2 (2%)	16 (12%)	111 (86%)
Information Sources	News	26 (17%)	13 (8%)	10 (7%)	104 (64%)
	Peer-reviewed articles	27 (18%)	8 (5%)	16 (11%)	96 (65%)
	Social media	10 (16%)	7 (11%)	3 (5%)	43 (68%)
	Family and/or friends	25 (18%)	7 (5%)	9 (6%)	98 (71%)
	Healthcare provider	26 (21%)	6 (5%)	8 (7%)	81 (67%)
	Other	4 (17%)	1 (4%)	2 (8%)	17 (71%)

**Table 3:** Themes related to vaccine hesitancy.

COVID-19 Vaccine Hesitancy Themes	Frequency (# of times theme was identified)
Perception of not being at risk	8
Mistrust	7
Concern of vaccine long-term effects	7
Prior COVID-19 infection	5
Concern of health risks	5
COVID-19 Vaccine Non-Hesitancy Themes	
Protecting myself and others	7
Moral obligation	6
Work or school related	5
A wish to return to normal	5
Influenza Vaccine Non-Hesitancy Themes	
Work or school related	3
Protecting myself and others	2
Trust in Influenza Vaccine	2
Trust in research process	2

## Data Availability

The data presented in this study are available on request from the corresponding author.

## Appendix A

### Appendix A.1. The FREQ Procedure, Student by Age

**Table A1:** Student by age.

Student (Student Status)	Age		
Frequency	18–26	27 and Older	Total
Undergraduate	124	8	132
Graduate	80	99	179
Total	204	107	311

**Table A2:** Statistics for table of student age.

Statistic	DF	Value	Prob
Chi-Square	1	81.6445	<0.0001
Likelihood Ratio Chi-Square	1	93.8879	<0.0001
Continuity Adj. Chi-Square	1	79.4769	<0.0001
Mantel–Haenszel Chi-Square	1	81.3820	<0.0001
Phi Coefficient		0.5124	
Contingency Coefficient		0.4560	
Cramer’s V		0.5124	

**Table A3:** Summary statistics: COVID-19 vaccination status (VX status) and hesitancy by age and student status.

		*n*					%				
		No	Will Wait	Will Receive	Have Received	Total	No	Will Wait	Will Receive	Have Received	Total
Undergraduate	18–26	36	16	9	63	124	29	13	7	51	100
	27 and older	6	1	1	.	8	75	13	13	.	100
	Total	42	17	10	63	132	32	13	8	48	100
Graduate	18–26	8	2	9	61	80	10	3	11	76	100
	27 and older	4	2	7	86	99	4	2	7	87	100
	Total	12	4	16	147	179	7	2	9	82	100
Total	18–26	44	18	18	124	204	22	9	9	61	100
	27 and older	10	3	8	86	107	9	3	7	80	100
	Total	54	21	26	210	311	17	7	8	68	100

**Table A4:** Analysis results: COVID-19 vaccination status (VX status) and hesitancy by age and student status.

Criterion	Value/DF	Global Test	Pr > Chi-Square		Type 3 Test			Pr > ChiSq
Deviance	1.7308	Likelihood Ratio	<0.0001		Age			0.0875
	.		.		Student			<0.0001
	.		.		Age student			0.0004
		Cumulative Odds Ratio (*p*-value) Comp. to		Cumulative Rate of VX Hesitancy ± SE				
Student Status	Age	27 and older		No		No, or will wait	No, will wait, or will receive	
Undergraduate	18–26	0.11(0.002)		27.9% ± 3.8%		38.8% ± 4.3%	51.1% ± 4.5%	
	27 and older			77.2% ± 14%		84.7% ± 10%	90.2% ± 7.1%	
Graduate	18–26	2.06(0.065)		10.1% ± 2.6%		15.5% ± 3.6%	23.3% ± 4.6%	
	27 and older			5.2% ± 1.6%		8.2% ± 2.3%	12.9% ± 3.3%	

**Table A5:** Summary statistics: COVID-19 vaccination status (VX status) and hesitancy by race and ethnicity.

	*n*					%				
	No	Will Wait	Will Receive	Have Received	Total	No	Will Wait	Will Receive	Have Received	Total
American Indian or Alaska Native	2	0	0	3	5	40	0	0	60	100
Asian	0	3	9	45	57	0	5	16	79	100
Black or African American	1	0	0	14	15	7	0	0	93	100
Hispanic or Latino	1	4	5	19	29	3	14	17	66	100
Native Hawaiian or Other Pacific Islander	0	0	0	0	0	.	.	.	.	.
White	48	14	13	135	210	23	7	6	64	100
Total	52	21	27	216	316	16	7	9	68	100

**Table A6:** Analysis results: COVID-19 vaccination status (VX status) and hesitancy by race and ethnicity.

Criterion	Value/DF	Global Test	Pr > Chi-Square	Type 3 Test	Pr > ChiSq
Deviance	1.8682	Likelihood Ratio	0.0025	q2_1	0.8744
	.		.	q2_2	0.0078
	.		.	q2_3	0.0024
	.		.	q2_4	0.0761
	.		.	q2_6	0.1219

### Appendix A.2. The FREQ Procedure by Annual Influenza Vaccine

**Table A7:** Student by Flu_vx.

Student (Student Status)				Flu_vx (Annual Influenza VX)						
Frequency				No		Yes		Total		
Undergraduate				92		39		131		
Graduate				86		87		173		
Total				178		126		304		
Frequency Missing = 7										
	Cumulative Odds Ratio (*p*-value) Comp. to						Cumulative Rate of VX Hesitancy ± SE			
Race	Asian	Black or African American	Hispanic or Latino		White		No		No, or will wait	No, will wait, or will receive
American Indian or Alaska Native	11.49 (0.045)	36.98 (0.016)	4.58 (0.191)		4.33 (0.204)		52.5% ± 28%		63.5% ± 26%	73.1% ± 22%
Asian		3.22 (0.220)	0.40 (0.079)		0.38 (0.003)		8.8% ± 2.7%		13.2% ± 3.7%	19.1% ± 4.9%
Black or African American			0.12 (0.023)		0.12 (0.007)		2.9% ± 2.9%		4.5% ± 4.5%	6.8% ± 6.6%
Hispanic or Latino					0.94 (0.895)		19.4% ± 6.5%		27.5% ± 8.2%	37.2% ± 9.5%
White							20.3% ± 2.8%		28.7% ± 3.2%	38.6% ± 3.5%

**Table A8:** Statistics for table of student by Flu_vx.

Student (Student Status)	Flu_vx (Annual Influenza VX)		
Frequency	No	Yes	Total
Undergraduate	92	39	131
Graduate	86	87	173
Total	178	126	304
Frequency Missing = 7			

Sample size = 304; frequency missing = 7.

**Table A9:** The FREQ procedure.

Table of Age by Flu_vx			
Age	Flu_vx (Annual Influenza VX)		
Frequency	No	Yes	Total
18–26	126	73	199
27 and older	52	53	105
Total	178	126	304
Frequency Missing = 7			

**Table A10:** Statistics for table of age by Flu_vx.

Statistic	DF	Value	Prob
Chi-Square	1	5.3880	0.0203
Likelihood Ratio Chi-Square	1	5.3583	0.0206
Continuity Adj. Chi-Square	1	4.8347	0.0279
Mantel–Haenszel Chi-Square	1	5.3703	0.0205
Phi Coefficient		0.1331	
Contingency Coefficient		0.1320	
Cramer’s V		0.1331	

Sample size = 304; frequency missing = 7.

### Appendix A.3. Summary Statistics

**Table A11:** Summary statistics: COVID-19 vaccination status (VX status) and hesitancy by annual influenza vaccination (VX) status.

	*n*					%				
	No	Will Wait	Will Receive	Have Received	Total	No	Will Wait	Will Receive	Have Received	Total
No	47	16	10	105	178	26	9	6	59	100
Yes	7	5	15	99	126	6	4	12	79	100
Total	54	21	25	204	304	18	7	8	67	100

**Table A12:** The LOGISTIC procedure.

Deviance and Pearson Goodness-of-Fit Statistics				
Criterion	Value	DF	Value/DF	Pr > ChiSq
Deviance	12.8902	2	6.4451	0.0016
Pearson	13.5442	2	6.7721	0.0011

Number of unique profiles: 2.

**Table A13:** Summary statistics: COVID-19 vaccination status (VX status) and hesitancy by influenza vaccination (VX) status, student status, and age.

			*n*					%				
			No	Will Wait	Will Receive	Have Received	Total	No	Will Wait	Will Receive	Have Received	Total
Undergraduate	18–26	No	34	12	4	34	84	40	14	5	40	100
		Yes	2	4	4	29	39	5	10	10	74	100
		Total	36	16	8	63	123	29	13	7	51	100
	27 and older	No	6	1	1	.	8	75	13	13	.	100
		Total	6	1	1	.	8	75	13	13	.	100
	Total	No	40	13	5	34	92	43	14	5	37	100
		Yes	2	4	4	29	39	5	10	10	74	100
		Total	42	17	9	63	131	32	13	7	48	100
Graduate	18–26	No	6	1	3	32	42	14	2	7	76	100
		Yes	2	1	6	25	34	6	3	18	74	100
		Total	8	2	9	57	76	11	3	12	75	100
	27 and older	No	1	2	2	39	44	2	5	5	89	100
		Yes	3	.	5	45	53	6	.	9	85	100
		Total	4	2	7	84	97	4	2	7	87	100
	Total	No	7	3	5	71	86	8	3	6	83	100
		Yes	5	1	11	70	87	6	1	13	80	100
		Total	12	4	16	141	173	7	2	9	82	100
Total	18–26	No	40	13	7	66	126	32	10	6	52	100
		Yes	4	5	10	54	73	5	7	14	74	100
		Total	44	18	17	120	199	22	9	9	60	100
	27 and older	No	7	3	3	39	52	13	6	6	75	100
		Yes	3	.	5	45	53	6	.	9	85	100
		Total	10	3	8	84	105	10	3	8	80	100
	Total	No	47	16	10	105	178	26	9	6	59	100
		Yes	7	5	15	99	126	6	4	12	79	100
		Total	54	21	25	204	304	18	7	8	67	100

**Table A14:** Analysis results: COVID-19 vaccination status (VX status) and hesitancy by influenza vaccination (VX) status, student status, and age.

Criterion			Value/DF		Global Test	Pr > Chi-Square	Type 3 Test	Pr > ChiSq
Deviance			1.8816		Likelihood Ratio	<0.0001	Student	<0.0001
			.			.	Age	0.2587
			.			.	Flu_vx	0.0669
			.			.	Age student	0.0048
			.			.	Student Flu_vx	0.0119
			.			.	Age Flu_vx	0.6824
				Cumulative Odds Ratio (*p*-value) Comp. to		Cumulative Rate of VX Hesitancy ± SE		
Student Status	Age	Annual Influenza VX		No		No	No, or will wait	No, will wait, or will receive
Undergraduate	18–26	Yes		0.18(<0.001)		10.1% ± 3.5%	16.2% ± 5.0%	24.5% ± 6.7%
		No				38.0% ± 5.1%	51.3% ± 5.4%	64.0% ± 5.1%
	27 and older	No				77.0% ± 14%	85.2% ± 10%	90.6% ± 6.8%
Graduate	18–26	Yes		0.99(0.979)		10.0% ± 3.6%	16.1% ± 5.2%	24.4% ± 7.0%
		No				10.2% ± 3.5%	16.3% ± 5.1%	24.7% ± 6.7%
	27 and older	Yes		1.37(0.603)		5.6% ± 2.2%	9.3% ± 3.3%	14.7% ± 4.8%
		No				4.2% ± 2.0%	7.0% ± 3.1%	11.2% ± 4.7%

**Table A15:** Summary statistics: The impact of previous COVID-19 infection on COVID-19 vaccine status (VX status) and hesitancy.

	*n*								%						
	No		Will Wait	Will Receive		Have Received		Total	No		Will Wait	Will Receive		Have Received	Total
No	41		18	21		180		260	16		7	8		69	100
Yes	13		3	4		24		44	30		7	9		55	100
Total	54		21	25		204		304	18		7	8		67	100
Criterion					Value/DF		Global Test			Pr > Chi-Square			Type 3 Test	Pr > ChiSq	
Deviance					0.2364		Likelihood Ratio			0.0377			test	0.0377	
		Cumulative Odds Ratio (*p*-value) Comp. to						Cumulative Rate of VX Hesitancy ± SE							
COVID-19 Positive		No						No			No, or will wait			No, will wait, or will receive	
Yes		1.97(0.038)						27.5% ± 6.0%			36.7% ± 6.9%			46.5% ± 7.3%	
No								16.2% ± 2.2%			22.7% ± 2.5%			30.6% ± 2.8%	

**Table A16:** Summary statistics: The impact of information sources on COVID-19 vaccine status (VX status) and hesitancy.

	*n*					%				
	No	Will Wait	Will Receive	Have Received	Total	No	Will Wait	Will Receive	Have Received	Total
News	26	13	10	104	153	17	8	7	68	100
Peer-reviewed articles	27	8	16	96	147	18	5	11	65	100
Social media	10	7	3	43	63	16	11	5	68	100
Family and/or friends	25	7	9	98	139	18	5	6	71	100
Healthcare provider	26	6	8	81	121	21	5	7	67	100
Other	4	1	2	17	24	17	4	8	71	100
Total	118	42	48	439	647	18	6	7	68	100

**Table A17:** Analysis results: The impact of information sources on COVID-19 vaccine status (VX status) and hesitancy.

Criterion	Value/DF		Global Test		Pr > Chi-Square			Type 3 Test		Pr > ChiSq	
Deviance	0.8866		Likelihood Ratio		0.9557			q7_1		0.7372	
	.				.			q7_2		0.8278	
	.				.			q7_3		0.9796	
	.				.			q7_4		0.3329	
	.				.			q7_5		0.7519	
	.				.			q7_6		0.5487	
	Cumulative Odds Ratio (*p*-value) Comp. to							Cumulative Rate of VX Hesitancy ± SE			
Source of Information	Peer-reviewed articles	Social media		Family and/or friends		Healthcare provider	Other	No	No, or will wait		No, will wait, or will receive
News	0.87(0.684)	0.91(0.838)		1.18(0.650)		0.85(0.644)	1.22(0.688)	18.2% ± 4.2%	25.3% ± 5.2%		33.6% ± 6.1%
Peer-reviewed articles		1.05(0.915)		1.35(0.353)		0.98(0.945)	1.40(0.507)	20.4% ± 4.3%	28.0% ± 5.2%		36.8% ± 5.9%
Social media				1.29(0.575)		0.93(0.867)	1.34(0.604)	19.6% ± 6.8%	27.1% ± 8.5%		35.8% ± 9.8%
Family and/or friends						0.72(0.359)	1.04(0.942)	15.9% ± 3.9%	22.3% ± 4.9%		30.1% ± 5.8%
Healthcare provider							1.44(0.487)	20.8% ± 5.1%	28.5% ± 6.2%		37.4% ± 7.0%
Other								15.4% ± 6.1%	21.7% ± 7.9%		29.4% ± 9.5%

**Table A18:** Summary statistics: The impact of negative health effects on COVID-19 vaccine status (VX status) and hesitancy.

	*n*					%				
	No	Will Wait	Will Receive	Have Received	Total	No	Will Wait	Will Receive	Have Received	Total
Self	4	2	3	17	26	15	8	12	65	100
Someone close	15	7	8	74	104	14	7	8	71	100
Someone close died	2	2	1	23	28	7	7	4	82	100
No negative health effect	33	13	15	114	175	19	7	9	65	100
Total	54	24	27	228	333	16	7	8	68	100

**Table A19:** Analysis results: The impact of negative health effects on COVID-19 vaccine status (VX status) and hesitancy.

Criterion			Value/ DF	Global Test		Pr > Chi-Square		Type 3 Test		Pr > ChiSq	
Deviance			1.3697	Likelihood Ratio		0.2380		q9_1		0.8769	
			.			.		q9_2		0.4295	
			.			.		q9_3		0.0907	
	Cumulative Odds Ratio (*p*-value) Comp. to						Cumulative Rate of VX Hesitancy ± SE				
Negative health impact	Someone close	Someone close died			No negative health effect		No		No, or will wait		No, will wait, or will receive
Self	1.31 (0.596)	2.41 (0.182)			1.07 (0.877)		20.6% ± 7.0%		28.3% ± 8.5%		37.3% ± 9.8%
Someone close		1.83 (0.316)			0.81 (0.429)		16.5% ± 3.4%		23.1% ± 4.2%		31.1% ± 4.9%
Someone close died					0.44(0.091)		9.7% ± 4.7%		14.1% ± 6.4%		19.8% ± 8.3%
No negative health effect							19.6% ± 2.7%		27.0% ± 3.2%		35.7% ± 3.5%

**Table A20:** Summary statistics: The impact of vaccine safety on COVID-19 vaccine status (VX status) and hesitancy.

	*n*					%				
	No	Will Wait	Will Receive	Have Received	Total	No	Will Wait	Will Receive	Have Received	Total
Not Confident	41	5	1	9	56	73	9	2	16	100
Undecided	9	8	2	35	54	17	15	4	65	100
Confident	4	8	22	160	194	2	4	11	82	100
Total	54	21	25	204	304	18	7	8	67	100

**Table A21:** Analysis results: The impact of vaccine safety on COVID-19 vaccine status (VX status) and hesitancy.

Statistic	Value	SE
Kendall’s Tau-b	0.5273	0.0473
Stuart’s Tau-c	0.4088	0.0443

**Table A22:** Summary statistics: The impact of vaccine efficacy on COVID-19 vaccine status (VX status) and hesitancy.

	The Impact of Vaccine Efficacy on COVID-19 Vaccine Status (VX Status) and Hesitancy.									
	*n*					%				
	No	Will Wait	Will Receive	Have Received	Total	No	Will Wait	Will Receive	Have Received	Total
Not Confident	33	5	1	9	48	69	10	2	19	100
Undecided	16	7	2	28	53	30	13	4	53	100
Confident	5	9	22	167	203	2	4	11	82	100
Total	54	21	25	204	304	18	7	8	67	100

**Table A23:** Analysis results: The impact of vaccine efficacy on COVID-19 vaccine status (VX status) and hesitancy.

Statistic	Value	SE
Kendall’s Tau-b	0.5213	0.0472
Stuart’s Tau-c	0.3930	0.0431

**Table A24:** Summary statistics: The impact of vaccine requirements on COVID-19 vaccine status (VX status) and hesitancy.

	*n*					%				
	No	Will Wait	Will Receive	Have Received	Total	No	Will Wait	Will Receive	Have Received	Total
No	54	16	4	53	127	43	13	3	42	100
Undecided	.	3	5	40	48	.	6	10	83	100
Yes	.	2	16	111	129	.	2	12	86	100
Total	54	21	25	204	304	18	7	8	67	100

**Table A25:** Analysis results: The impact of vaccine requirements on COVID-19 vaccine status (VX status) and hesitancy.

Statistic	Value	SE
Kendall’s Tau-b	0.4525	0.0419
Stuart’s Tau-c	0.3805	0.0396
